# Fibroids and Pregnancy

**Published:** 1901-06-22

**Authors:** 


### FIBROIDS AND PREGNANCY.

At the last meeting of the Obstetrical Society Dr. Archibald Donald read a paper on Fibroid Tumours of Complicating Pregnancy and Labour, which led to a considerable discussion. Dr. Donald said that he had come to the conclusion that in the majority of instances neither pregnancy nor labour was seriously influenced by these tumours, but that in a small proportion of cases the danger to mother and child was greatly increased. In regard to the treatment of this complication two main groups were to be recognised, first those in which pregnancy was allowed to take its course until full term or until the child was viable, and second those in which it was necessary to interfere in the earlier months. In the great majority of cases it was best he thought to leave matters alone until term, but if the tumour were situated in the pelvis the treatment must be settled by a careful examination—if necessary under an anæsthetic. Cæsarian section was preferable to delivery by the natural passages if the latter involved much force, and he related a case in which under such conditions Cæsarian section, followed by hysterectomy, was successfully employed. In regard to the treatment which might be required in the earlier months he thought that induction of abortion ought to be abandoned, one of the reasons being that in the sort of cases in which it might seem to be required its performance was attended with increased risk, partly from difficulty with the placenta, and partly from increased danger of sepsis. In cases of rapidly-growing fibroids in early pregnancy he advocated a preliminary myomectomy wherever this was possible, and thought that this ought to be done in all cases of subperitoneal pedunculated fibroids, while when sessile tumours required removal they might be enucleated and the gap in the uterus stitched up. The surgeon must, however, be prepared if necessary to terminate his operation by hysterectomy. In the discussion there seemed to be a considerable variety of opinion on many points, but there was practical unanimity upon one—namely, that on the discovery of a fibroid tumour which seems likely to interfere with the progress of pregnancy or labour it is not a wise thing to induce abortion. The president (Dr. Horrocks) was indeed the only one who had a word to say for it, holding that whilst it was true that with modern aseptic methods the mortality of hysterectomy had been greatly reduced the same was true in regard to the induction of miscarriage. Nevertheless, he said that aseptic methods had not eliminated one danger in the induction of miscarriage from fibroids, and that was hæmorrhage. This danger he said was considerable, for it was often impossible, owing to the increased length and tortuosity of the canal, to reach with the finger so as to peel off the whole of the placenta. Another point which was pretty strongly insisted on by several speakers was that in a far larger number of cases than was by some supposed to be the case, pregnancy and labour were safely got through, notwithstanding the presence of fibroids, even although in the earlier months it might have seemed that their existence was likely to cause obstruction. Mr. Bland Sutton went so far as to say that next to pregnancy it seemed probable that fibroids were one of the commonest conditions to which a woman's uterus was liable. As proving how often the complication of pregnancy with fibroids may lead to no serious harm, and as showing the impropriety of inducing artificial miscarriage to avert mischief which may never happen, Dr. Herbert Spencer stated that at University College Hospital, where about 2,500 cases of labour were attended annually, he had seen a considerable number of cases of pregnancy and labour complicated with fibroid tumours, but he had never met with a death from the complication, and had only once had to perform Cæsarian section and to remove the uterus. He had seen cases he said in which it was expected that fibroids in the lower segment would interfere with the passage of the child, but when labour set in the tumours were drawn up out of the way, and delivery was safely effected. He thought that the statement that all subperitoneal pedunculated fibroids ought to be removed during pregnancy was too absolute, and he entered a protest against the removal on account of pain of small sessile corporeal fibroids by enucleation through the abdomen during pregnancy.

